# Unexplained Fatal Hyperammonemia in a Patient With New Diagnosis of Acute Monoblastic Leukemia

**DOI:** 10.7759/cureus.20108

**Published:** 2021-12-02

**Authors:** Mohamed Fayed, Nimesh Patel, Yahia Al Turk, Patrick B Bradley

**Affiliations:** 1 Anesthesiology, Pain Management and Perioperative Medicine, Henry Ford Health System, Detroit, USA; 2 Internal Medicine, Henry Ford Health System, Detroit, USA; 3 Pulmonary and Critical Care, Henry Ford Health System, Detroit, USA

**Keywords:** general nephrology dialysis and transplantation, chemo radiotherapy (chemo-rt), supportive and palliative care, brain herniation, serum ammonia, ammonia, acute myeloid leukemia (aml), leukemia

## Abstract

Idiopathic hyperammonemia is a serious condition that can arise after induction of chemotherapy and is characterized by plasma ammonia levels greater than two times the normal upper limit but within the context of normal liver function. While this dangerous complication usually appears several weeks after the start of chemotherapy, we report a fatal case of idiopathic hyperammonemia that was detected only nine days after induction chemotherapy in a 22-year-old man with no liver pathology or other risks for hyperammonemia. The patient’s initial emergent presentation was altered mental status. Laboratory workup showed acute monoblastic leukemia and radiological investigation showed cerebral hemorrhagic foci secondary to leukostasis. He received leukoreduction apheresis and he was started on induction chemotherapy with daunorubicin and cytarabine. On the ninth day of induction chemotherapy, it was noted that he developed worsening neurological findings. Investigations showed significant elevation in ammonia level and associated cerebral edema. Although hyperammonemia was mitigated, the patient’s cerebral status worsened and he died 15 days after initial presentation. This case shows that critical hyperammonemia can occur quickly after chemotherapy induction and that strategies for preventing a rise in plasma ammonia are necessary.

## Introduction

Hyperammonemia can occur as a complication of a diverse range of disorders including liver failure and inborn errors of urea metabolism [1]. Idiopathic hyperammonemia has been defined as a plasma ammonia level greater than two times the upper normal limit (normal range: 18-50 µmol/L) with relatively normal liver functions [2,3].

The first reported cases of idiopathic hyperammonemia after induction of chemotherapy for acute leukemia were described in the 1980s [4]. This complication typically appears around two weeks after the start of chemotherapy [4-7]. Prognosis of idiopathic hyperammonemia in patients with hematologic malignancies is poor and with mortality rates as high as 80% in one case series [5].

We report the case of a young male patient who developed severe fatal hyperammonemia that started nine days after starting chemotherapy. A case with such rapid onset and degree of severity is yet to be described in the literature. Despite aggressive treatment, his condition was complicated with diffuse cerebral edema ultimately leading to his death.

## Case presentation

A 22-year-old man with no significant medical history was brought into the emergency department by his mother because of altered mental status. During the previous week, he had experienced bilateral dull aching headache associated with fever, blurry vision, nausea, and vomiting. Examination revealed a diaphoretic pale-looking young man. Vital signs showed tachycardia, low oxygen saturation, and fever. His Glasgow Coma Scale was 14 out of 15, he was oriented only to persons.

Initial laboratory workup showed complete blood count and film evidence of leukocytosis (WBC 395 K/µL) with 94% blasts, hemoglobin 6.2 g/dL, and platelet count 61 K/µL. The patient’s liver function tests revealed alanine transaminase (ALT) of 724 IU/L, aspartate aminotransferase (AST) 741 IU/L, and normal bilirubin at 1.1 mg/dL. Computed tomography (CT) brain showed multiple bilateral hemorrhagic foci with surrounding vasogenic edema. MRI of the brain confirmed hemorrhagic foci and it didn't show evidence of meningeal involvement of leukemia. Bone marrow biopsy confirmed monoblastic leukemia.

He was started on empirical antibiotics of vancomycin, cefepime, and voriconazole. He received induction chemotherapy with daunorubicin (60 mg/m^2^ per day for three days) and cytarabine (100 mg/m^2^ per day for seven days). Leukoreduction apheresis was done for leukostasis as leukocytosis was believed to contribute to his cerebral hemorrhagic foci. His liver functions normalized within seven days (Table 1). Cerebrospinal fluid sampling was not done as the patient was coagulopathic with pancytopenia at the earlier stage of treatment.

**Table 1:** Trend in liver enzymes and liver function tests after induction chemotherapy. ALT: alanine transaminase; AST: aspartate aminotransferase; IU: international unit

Marker	Normal range	Day 1	Day 2	Day 3	Day 4	Day 5	Day 6	Day 7	Day 8
ALT	<52 IU/L	534	537	317	290	96	84	58	44
AST	<35 IU/L	420	435	294	26	75	49	33	35
Albumin	3.7-4.8 g/dL	2.3	2.0	3.1	3.0	3.7	3.2	3.1	3.0
Protein, total	6.0-8.3 g/dL	4.5	4.1	5.5	5.6	6.6	6.2	6.1	5.9
Bilirubin, total	<1.2 mg/dL	0.8	0.7	0.5	0.7	0.7	0.8	0.8	0.8
Bilirubin, direct	0 -0.3 mg/dL	0.4	0.2	0.2	0.3	0.2	0.3	0.3	0.3
Alkaline phosphatase	40-140 IU/L	61	53	51	54	57	51	46	47

**Figure 1:**
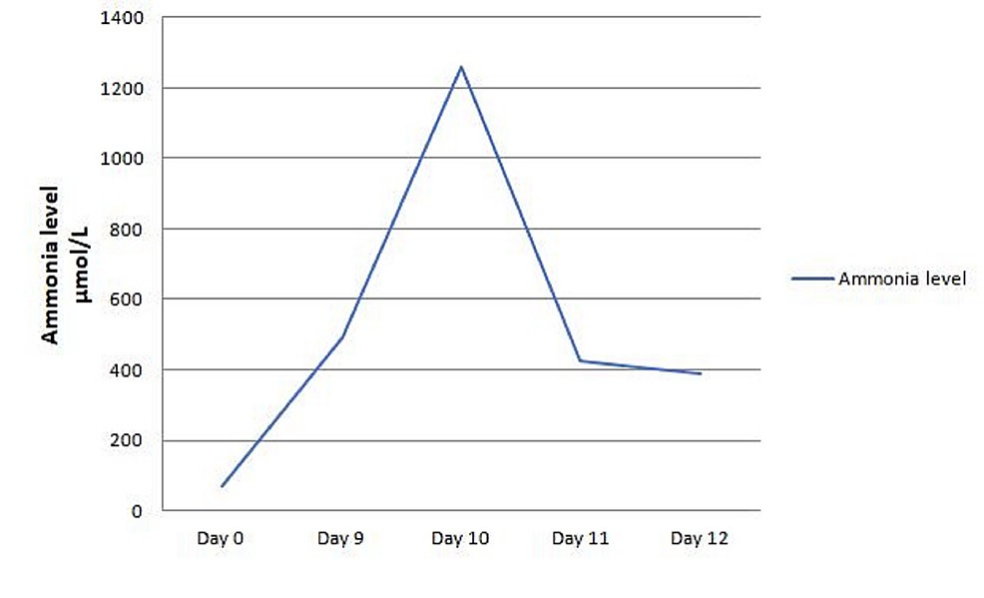
Patient’s ammonia levels during hospitalization.

On day nine after the start of chemotherapy, neurological examination showed decerebrate posture. Laboratory results showed significant elevation in ammonia levels that peaked at 1262 µmol/L (normal range: 18-50 µmol/L) (Figure 1). Ultrasound of the abdomen showed that his liver surface contour and echogenicity of the liver were within normal limits with no evidence of thrombi in the hepatic and portal veins. Hepatitis markers were negative for hepatitis A, B, and C viruses. Transthoracic echocardiogram showed normal left and right ventricular functions with pulmonary artery pressure of 34 mmHg.

Considering the findings, multidisciplinary team including hepatologist, intensivist, hematologist, and nephrologist believed that the patient's high ammonia level (>1000 µmol/L) was not related to any intrinsic liver disease. Possible etiologies include ammonia production from excessive amino acid catabolism secondary to chemotherapy (most likely), urea cycle enzyme defect (patient never had a history of symptoms and signs of high urea level), acute liver failure (synthetic functions of the liver were normal), and sepsis or infection from urease producing organisms (repeated blood cultures were negative).

He was started on neuroprotective measures including controlling fever with active cooling, head elevation, and osmotherapy with targeting serum sodium 150-155 mEq/L. He was provided adequate nutritional support via orogastric tube. He started on oral lactulose (20 g six times per day) aiming for three soft bowel motions per day and oral elemental zinc (50 mg per day). Nephrology team was consulted and they initiated continuous veno-venous hemofiltration (CVVHF), using 5 L as replacement fluids and targeting replacement fluid sodium concentration of 155 mEq/L. Neurosurgical team was consulted and they had little to offer in this case, including surgical intervention to relieve intracranial pressure or insertion of specified catheter to monitor intracranial pressure, this is because he had high risk of bleeding and infection.

**Figure 2:**
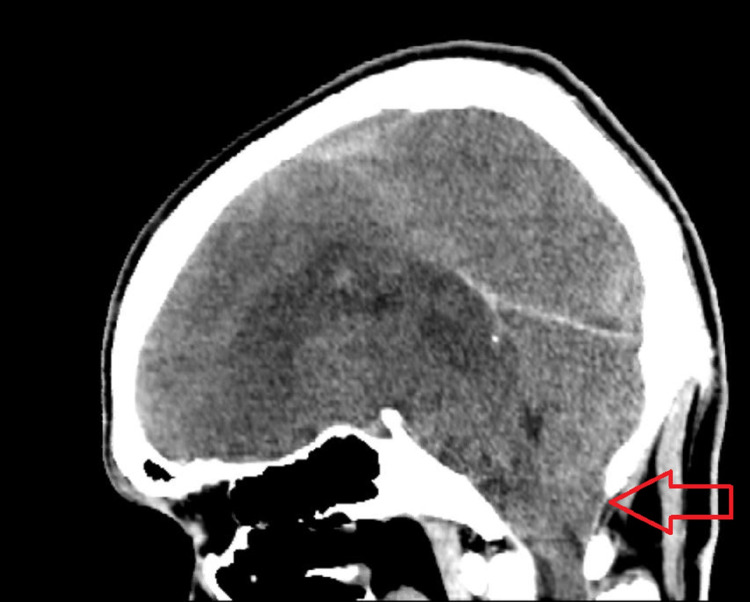
Computed tomography, sagittal section, showing evidence of cerebellar tonsillar herniation through foramen magnum. (Red arrow showing the level of the foramen magnum.)

Despite treatment for hyperammonemia that resulted in a decline in the ammonia level to 388 µmol/L, his neurological status continued to deteriorate (Figure 1). Repeated examination showed bilateral dilated pupils, absent corneal reflexes, and absent spontaneous respiratory efforts. A repeated CT brain showed worsening effacement of the cerebral sulci and basal subarachnoid cisterns, with new tonsillar herniation (Figure 2). Goals of care were discussed with the patient's family, they decided to stop all treatment and to proceed with comfort care measures only. The patient died 15 days after his initial presentation.

## Discussion

Here, we presented the case of a patient who developed severe hyperammonemia nine days after induction of chemotherapy with cytarabine and daunorubicin, which led to tonsillar herniation and brain death. The incidence of idiopathic hyperammonemia after chemotherapy may be as high as 2.4% [1]. Previously reported cases of idiopathic hyperammonemia have shown that this complication usually occurs several weeks after the start of chemotherapy, and it is usually not as severe as what our patient experienced [5,6].

The physiological mechanism of hyperammonemia is likely multifactorial. Cytarabine contributes slightly to ammonia formation through a metabolic process of deamination, however, with an extremely high number of blast cells such as what we observed in our patient (white blood cells 395 K/µL with 94% blasts), deamination can lead to even higher ammonia levels [7]. Our patient had an initial elevation of transaminase enzymes from sepsis that could have contributed to altered hepatic amino acid metabolism [8]. There is insufficient evidence to verify that a congenital metabolic disorder or urea cycle disorder had contributed to hyperammonemia in our patient as he was a healthy young man; as it usually presents during infancy and childhood therefore, however, heterozygous carrier of the enzymatic disorder might present under stressful conditions like this case [9]. We did not investigate a urea cycle defect by measuring citrulline, ornithine, or arginine levels for several reasons, as this would not have changed our management plan, the test results would have taken few days to weeks to come back, and interpretation of these results in presence of hemodialysis could be challenging. Our patient didn’t have constipation, as there have been reported cases of hyperammonemia secondary to the microbial proliferation of urease-producing bacteria such as Proteus species and he did not have a history of taking drugs that could have been a potential cause of his hyperammonemia [10]. Although his liver function was not optimal during the initial days of chemotherapy, it had improved after the hyperammonemia had been detected and treated. Furthermore, we didn’t observe evidence of hepatic failure among the other relevant laboratory data.

**Figure 3:**
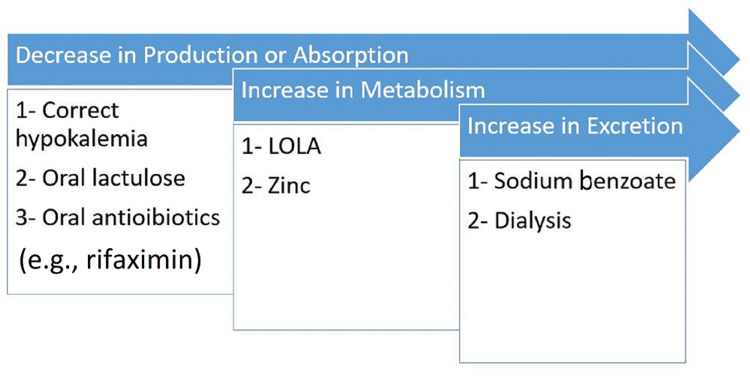
Strategies of controlling hyperammonemia. LOLA: L-ornithine-L-aspartate

Several strategies have been proposed for controlling hyperammonemia [11] (Figure 3). Decrease in ammonia production and absorption, increase in ammonia metabolism, and increase in ammonia elimination from bloodstream via dialysis or medications, e.g., sodium benzoate. Decrease in ammonia production and absorption is achieved by correcting hypokalemia, administering oral lactulose, and/or oral antibiotics (e.g., rifaximin or neomycin) that lead to colonization of non-urease producing bacteria. Hypokalemia leads to increase in renal tubular ammonia production [12]. Lactulose is metabolized by the flora and leads to acidic pH that leads to non-absorbable ammonium that is trapped in the colon [13,14]. Drugs that increase ammonia metabolism into urea include L-ornithine-L-aspartate (LOLA) and zinc. LOLA stimulates carbamoyl phosphate synthetase and ornithine-transcarbamylase (OTC) that are key enzymes in the urea cycle, zinc may modulate ion channel function and neurotransmission [15] and it stimulates OTC enzyme [16]. An increase in the elimination of ammonia can be achieved by pharmacological agents (sodium benzoate) or dialysis. Sodium benzoate has been proposed to promote the elimination of ammonia by reacting with glycine to form hippurate (renally excreted), however, this has variable outcomes [17]. Intermittent hemodialysis or CVVHF can be used to eliminate urea. It has been suggested that dialysis should be started early in the progression to hyperammonemia and before the development of acute kidney injury so that the concentration of ammonia will not rise to a clinically significant level [18]. Continuation of hemodialysis until the blood ammonia concentration has dropped below 200 μmol/L for a period of at least 24 hours is generally suggested. CVVHF may seem to be a less effective strategy than hemodialysis but it is a continuous method that can be maintained for a longer period and can clear newly produced nitrogen [19]. The molecular adsorbent recirculating system is a technique based upon albumin dialysis. It promotes the elimination of bilirubin, bile acids, and ammonia [20]. It has been used with some efficacy in case of L-asparaginase-related hyperammonemia [6]. In our patient, we used oral lactulose, zinc supplement, and we started him on CVVHF. We chose CVVHF over intermittent hemodialysis, as we wanted to use it for longer period of time and gradual decrease in ammonia level compared to hemodialysis. Other above-mentioned medications such as sodium benzoate, LOLA, and albumin dialysis were not available in our institution.

## Conclusions

Hyperammonemia can present after starting chemotherapy in patients with acute leukemia. Hence it is important for prevention and early recognition by checking serial ammonia levels after starting chemotherapy. One should consider starting at lower dose or delaying commencement of chemotherapy in patients with multiorgan failure, critically unstable, or with significantly elevated blast count like in our patient.
